# Brazilian Urologist's mental health aspects auring the Covid-19 pandemic

**DOI:** 10.1590/S1677-5538.IBJU.2020.0869

**Published:** 2020-12-20

**Authors:** Aline Gularte Teixeira da Silva, Johanna Ovalle Diaz, Antônio Rebello Horta Görgen, Victor Hugo Schwengber, Renan Timoteo de Oliveira, Patric Machado Tavares, Tiago Elias Rosito

**Affiliations:** 1 Hospital de Clínicas de Porto Alegre Serviço de Urologia Porto AlegreRS Brasil Serviço de Urologia, Hospital de Clínicas de Porto Alegre, Porto Alegre, RS, Brasil.

## INTRODUCTION

Burnout is a psychological syndrome that emerges in response to emotional and interpersonal work-related stressors. Its main manifestations are exhaustion, depersonalization, and inefficiency (1). The prevalence of burnout among physicians is high, with an estimated percentage of 43.9% and 41.7% of physicians with a positive depression screen (2). Burnout rates among American urologists are also high with 38.8% meeting burnout criteria (3).

The following factors are associated with a higher prevalence of burnout among physicians: female gender, young age, long work hours, low job satisfaction, and conflicts between professional and personal life (4). Other factors that may also influence the development of burnout syndrome in physicians are chronic stress, lack of autonomy in the work environment and sleep deprivation (5). As with burnout, the prevalence of sleep deprivation and circadian disorders is high among physicians, and their health-related effects are similar (6). A positive screen for sleep disorders is associated with an approximately four-fold increase in burnout rates (7).

Burnout negatively affects patient care and healthcare systems, decreasing patient satisfaction, increasing medical errors and job switching, and leading to early retirements (8). It permeates urologist's culture from the beginning of their training. It persists as a significant factor for work dissatisfaction, interpersonal conflicts, and psychoactive substance abuse, even in highly experienced professionals (9).

On January 9^th^, 2020, the World Health Organization (WHO) reported that an unprecedented coronavirus strain (Sars-CoV-2) caused the outbreak of viral pneumonia, Covid-19, in Wuhan, China. On January 30^th^, 2020, with 98 confirmed cases of the disease in 18 countries other than China, WHO Director-General declared the global outbreak of Covid-19 a public health emergency of international concern (10). In Brazil, the first confirmed Sars-CoV-2 infection case was on February 26^th^ in the state of São Paulo. By the time of this review, Brazil had more than 4.528.756 confirmed cases and 136.575 deaths.

The Covid-19 pandemic represents one of the most significant challenges in modern medicine (11). Healthcare professionals, who are directly involved in the diagnosis, treatment, and care of patients with Covid-19, are at a higher risk of developing psychological stress and other mental health symptoms (12). The aspects related to the psychosocial impacts of Covid-19 on health professionals are feelings of helplessness, guilt, distancing from family during quarantine, exhaustion, depression, fear of infection and complications, uncertainty, overworking, and substance abuse (13). The acute worsening of systemic stressors may lead to a significantly negative impact on the healthcare system and patient safety (14). As with other specialties, the Covid-19 pandemic dramatically changed the urologist's routine (15).

This study aimed to identify the rates of burnout, stress, and sleep disorders among Brazilian urologists during the Covid-19 pandemic.

## MATERIALS AND METHODS

This study was approved by the Ethics Committee of the Porto Alegre Clinical Hospital (CAAE 31645020.5.0000.5327).

Brazilian urologists practicing during the Covid-19 pandemic and six months prior were eligible for the study. An anonymous questionnaire on the Google Forms platform was sent to Brazilian urologists on July 1^st^, 2020, and each urologist was asked to send it to their teammates. The physicians agreed to the informed consent form before filling the questionnaire. We closed collections on July 30^th^, 2020.

We developed the questionnaire to assess the prevalence of burnout, stress, and poor sleep quality among Brazilian urologists during the pandemic. It consisted of demographic and clinical practice questions (gender, the region of the country where they work, work institution, number of urologists on the team, working hours, and surgical procedure hours), and the following questionnaires: Copenhagen Burnout Inventory (CBI), Perceived Stress Scale - 10 (PSS-10), and Pittsburgh Sleep Quality Index (PSQI).

We assessed burnout using the CBI, which consisted of 19 questions divided into three domains: personal burnout, work-related burnout, and patient related burnout. We considered burnout significant when the score was greater than or equal to 50% in each domain or on average. We assessed stress perception using the PSS-10, which consisted of ten questions and whose final measure is classified as low (0-13), moderate (14-26), or high (27-40). We assessed sleep quality through the PSQI, which comprised seven components. When the sum was greater than five, we considered this to indicate poor sleep quality.

We assessed the association between qualitative variables using the Fisher's exact test. We used the Mann-Whitney U test for independent samples with two categories to compare the means, and the Kruskal-Wallis test for samples with more than two categories. We tabulated the data in a Microsoft Office Excel spreadsheet and analyzed it using the IBM Statistical Package for the Social Sciences (v.20.0). The significance level was 5% (p <0.05).

## RESULTS

A total of 165 urologists answered the questionnaire, namely 127 men (77%) and 38 women (23%). Physicians from throughout Brazil participated, with a predominance of the Southern region (65.5%, or 108), followed by the Southeastern region (24.8%, or 41). Out of all questionees, 52.8% (87) work in an academic institution, and 45.5% (75) work exclusively in non-academic institutions. Additionally, 83.9% (125) work in teams with other urologists, 67.9% (112) spent less than 20 hours a week in hospitals, and 48.5% (80) dedicated less than six hours a week to surgical practice during the pandemic.

**Figure 1:**
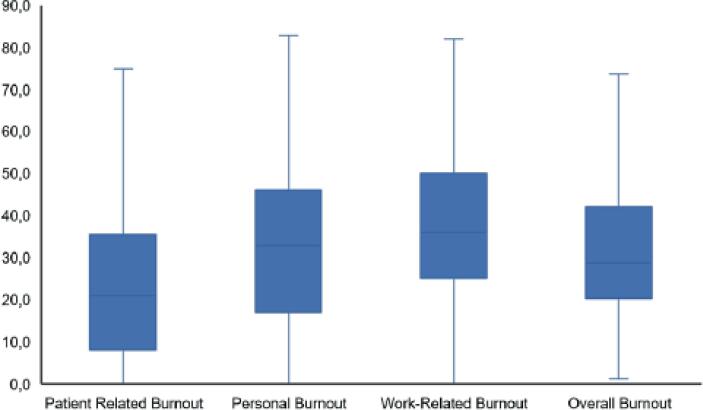
Boxplot - Brazilian urologists’ burnout rates during the Covid-19 pandemic

Regarding the CBI results, 7.87% (13) experienced patient-related burnout, 23% (38) work-related burnout, and 20.6% (34) personal burnout. When we calculated the average between the three domains, the overall burnout prevalence was 15.5% (25) (Figure-1). In females, there was a higher statistically significant level of personal burnout (p=0.005) and work-related burnout (p=0.001) in the bivariate analysis, with no significance in patient-related burnout.

Regarding the PSS-10 results, 57.57% (95) of the urologists showed a moderate to high perceived stress during the pandemic, with a significant association with gender (p=0.043): 73.68% (28) of women and 52.75% (67) of men had a moderate to high perceived stress.

Regarding the PSQI results, 44.84% (74) of the urologists had a poor sleep quality, with 60.5% (15) of women and 46.45% (59) of men, with no significant difference between genders.

There was a significant association between sleep quality and overall burnout (p=0.008) (Table-1), work-related burnout (p=0.009), and personal burnout rates (p <0.001). The same association occurred between the perceived stress levels and overall burnout (p <0.001), work-related burnout (p <0.001), and personal burnout rates (p <0.001).

**Table 1:** Burnout distribution in relation to sleep quality and perceived stress level of Brazilian urologists during the COVID-19 pandemic

	Burnout				p-value
	No		Yes		
	n	%	n	%	
**Sleep Quality**					
Poor	69	(49,3)	5	(20)	0,008
Good	71	(50,7)	20	(80)	
**Perceived Stress Level**					
Low	67	(47,9)	3	(12)	<0,001
Moderate	67	(47,9)	13	(52)	
High	6	(4,3)	9	(36)	

**p** = Fisher's Exact test

## DISCUSSION

Ours is the first study that evaluated burnout, stress and sleep disorders among Brazilian urologists. Burnout rates in urology gained prominence when Dr. Shanafelt published an update to his 2011 study with 2014 data, demonstrating a significant increase in burnout rates - which were already high - among urologists over this period (greater than 20%) (16). According to his data, derived from a subgroup analysis with 119 urologists (1.7% of the sample), the prevalence of burnout in this specialty was 63.6% in 2014. In 2017, the most extensive study to evaluate burnout in urologists took place using data derived from the 2016 American Urological Association (AUA) census, determining an overall burnout prevalence of 38.8% among American urologists (3). Our study, the first carried out with urologists during the Covid-19 pandemic, showed a prevalence of 23% work-related burnout, 20.6% personal burnout, and 7.87% patient-related burnout. After calculating the average between the three domains, the overall prevalence was 15.5%.

The rates found in our study are lower than those previously published with U.S. data (2, 3), even being collected during a pandemic. This may be explained by the decrease in the urologist's working time in hospitals and the lesser number of elective surgeries, with less worked hours - a factor strongly associated with the prevalence of burnout among Brazilian doctors. This decrease occurred due to a recommendation to reschedule elective surgeries during the pandemic to avoid the unnecessary exposure of patients and surgical staff and avoid the excessive use of personal protective equipment (15). It was suggested the treatment for urgent or emergent urological conditions only, even face to face and diagnostic activities underwent a great reduction, and in some cases a complete cancellation (17). It was also recommended that outpatients diagnostic procedures should be postponed, especially those requiring general anesthesia or sedation (18). Our findings confirm that the workload is a risk factor for burnout.

When comparing the average levels of burnout between men and women, our study found significantly higher personal burnout (p=0.005) and work-related burnout (p=0.001) in women. When assessing stress levels, women also had significantly higher levels of stress than men (p=0.043). These findings are compatible with previous publications that indicate higher levels of burnout among women in surgical specialties, confirming that the female gender is a risk factor for burnout among physicians. However, the study carried out with American urologists in the AUA census does not present the same association (3). Among the possible reasons for higher stress levels and personal burnout among women is the conflict between home and work life. Schools and daycare centers remained closed during the pandemic, increasing the time of child care at home. Another crucial aspect is that the proportion of female urologists is lower in Brazil than in the U.S. In Brazil, women accounted for only 2% (126) of the total number of members of the Brazilian Society of Urology in 2018. However, in the U.S., women represent 9.9% of urologists associated with AUA. Our study evaluated 30% of Brazilian female urologists (38/126).

In our sample, 74/165 (44.84%) of the urologists had poor sleep quality during the pandemic, with no significant difference between genders. We found an association between sleep quality and levels of overall (p = 0.008), work-related (p = 0.009), and personal (p <0.001) burnout. This finding is consistent with studies showing that poor sleep quality is a risk factor for burnout (7, 8).

Regarding sleep disorders, 44.84% (74) of urologists had poor sleep quality and 57,57% (95) of urologists had moderate to high perceived stress levels. Additionally, we also found an association between stress levels and overall (p <0.001), work-related (p <0.001), and personal burnout (p <0.001) levels. The individual's control under daily life circumstances impacts their perceived stress. During a pandemic, there is a decrease in the predictability of events, especially among health professionals, who had their work routines significantly changed, including the telemedicine's emergency implementation to maintain urological care quality and avoid the risk of disseminating Covid-19 (19).

A positive aspect of our study is that it is the first of its kind to assess aspects of mental health in Brazilian urologists. Despite the limitations of a survey study, we obtained a representative sample of Brazilian urologists working during the pandemic. Additionally, it is the first study to evaluate burnout, stress and sleep disorders in urologists during the pandemic.

## CONCLUSIONS

Burnout prevalence in Brazilian urologists during the Covid-19 pandemic was lower than previous U.S findings but consistent with the burnout rates among Brazilian physicians. We found a high level of stress and poor sleep quality and also a significant association between stress and poor sleep quality with burnout levels among urologists.
